# Melatonin is responsible for rice resistance to rice stripe virus infection through a nitric oxide-dependent pathway

**DOI:** 10.1186/s12985-019-1228-3

**Published:** 2019-11-21

**Authors:** Rongfei Lu, Zhiyang Liu, Yudong Shao, Feng Sun, Yali Zhang, Jin Cui, Yijun Zhou, Wenbiao Shen, Tong Zhou

**Affiliations:** 1 College of Life Sciences, Laboratory Center of Life Sciences, Nanjing Agricultural University, Nanjing, 210095 China; 2 Key Laboratory of Food Quality and Safety, Institute of Plant Protection, Jiangsu Academy of Agricultural Sciences, Nanjing, 210014 Jiangsu Province China; 3 College of Resources and Environmental Sciences, Nanjing Agricultural University, Nanjing, 210095 China; 4 School of the Environment and Safety Engineering, Jiangsu University, Zhenjiang, 212013 Jiangsu Province China; 5 International Rice Research Institute and Jiangsu Academy of Agricultural Sciences Joint Laboratory, Nanjing, 210095 China

**Keywords:** Melatonin, Nitric oxide, *Rice stripe virus*, Rice

## Abstract

*Rice stripe virus* (RSV) causes one of the most important rice virus diseases of plants in East Asia. However, the molecular mechanisms controlling rice resistance to RSV infection are largely unknown. Recently, several studies presented a novel model that melatonin (MT) and nitric oxide (NO) participate in the plant-pathogen interaction in a synergetic manner. In this study, there was a difference in MT content between two rice varieties that correlated with one being susceptible and one being resistant to RSV, which suggested that MT is related to RSV resistance. In addition, a test with two NO biosynthesis inhibitors revealed that NO inhibitor were able to increase the disease incidence of RSV. A pharmacological experiment with exogenous MT and NO showed that increased MT and NO in the MT-pretreated plants led to lower disease incidences; however, only NO increased in a NO-releasing reagent [sodium nitroprusside (SNP)] pretreated plants. The expressions level of *OsPR1b* and *OsWRKY 45* were significantly induced by MT and NO. These results suggest that rice resistance to RSV can be improved by increased MT through a NO-dependent pathway.

## Introduction

*Rice stripe virus* (RSV), a typical member of the genus *Tenuvirus*, is transmitted mainly by the small brown planthopper (*Laodelphax striatellus*) (SBPH) in a persistent and propagative manner, and can infect many crops, such as rice, wheat, and several other gramineous plants, resulting in a severe loss of grain [1]; in addition, Sun et al. reported that the virus can also infect *Arabidopsis thaliana* [2]. The infected plants exhibit typical symptoms, such as alternating yellow and green stripes, curly and drooping leaves, and necrotic lesions [3]. Previous reports indicated that the plant chloroplasts were damaged by excessive accumulation of starch, which was triggered by RSV infection, resulting in alternating yellow and green stripes [4]. Melatonin (N-acetyl-5-methoxytryptamine, MT), an amine hormone that was isolated and identified from the pineal gland in the late 1950s, has been revealed to possess a broad spectrum of biological functions in both animals and plants in many studies [5–9]. In particular, MT has been considered as a therapeutic indole for combating viral diseases, such as SARS (*severe acute respiratory syndrome*) and WNV (*West Nile virus*) [10]. Consistent with the benefits of MT in immunology and medicine in animals, MT can also induce plant resistance to pathogens, such as *Pseudomonas syringae DC3000*, *Alternaria spp.*, and *Fusarium spp.* [11, 12]. However, the detailed mechanisms of MT -triggered plant innate immunity to viruses are elusive. Recently, several studies also indicated the potential involvement of a gaseous signal molecule or plant hormone in the defense response triggered by MT. For example, it was reported that nitric oxide (NO) induced by MT is responsible for disease resistance to *Pst DC3000* infection in *Arabidopsis* [13]. Indeed, the vital role of NO in plant-pathogen interactions has been demonstrated by many studies [14–18]. NO can be produced through the nitrate/nitrite-dependent pathway that is known to be catalyzed by nitrate reductase (NR) [19] and through the _L_-arginine-dependent pathway that is known to be catalyzed by a mammalian NO synthase (NOS)-like enzyme [20, 21]. Further, Lee et al. found that the disease resistance induced by MT correlates with plant hormones in *Pst DC3000* [11, 22]. Thus, these studies may provide a potential mechanism for the effect of MT and NO in plant against other pathogen infections.

In this study, the endogenous MT level was monitored and quantified in rice after RSV infection. Further, the link among MT, NO and resistance genes was investigated.

## Materials and methods

### Chemicals

Unless otherwise stated, all chemicals used in this study were purchased from Sigma-Aldrich (Sigma-Aldrich, St Louis, MO, USA). In this study, sodium nitroprusside (SNP) was used as a NO-releasing reagent, diluted to 100 μM in water, and it was applied to soil in pots (9 cm in diameter and 14 cm tall, 25 mL/pot) with rice seedlings. The old SNP solution was obtained as a negative control by maintaining a 100 μM SNP solution for at least 5 days in the light in an open tube to eliminate NO as described [23, 24]. 2-(4-carboxyphenyl)-4, 4, 5, 5-tetramethylimidazoline-1-oxyl-3-oxide (cPTIO), a NO specific scavenger, was diluted to 100 μM in water prior to use. In addition, 200 μM NG-nitro-L-Arg methyl ester hydrochloride (L-NAME; a nitric oxide synthase (NOS) inhibitor) and 200 μM tungstate (a nitrate reductase (NR) inhibitor), were applied.

### Plant materials, virus isolates and inoculation assay

Rice (*Oryza sativa L. cv.*) seeds of Nipponbare and Zhendao 88, which are susceptible and resistant to RSV, respectively, were both obtained from the Jiangsu Academy of Agricultural Science (JAAS), Jiangsu, China. The viruliferous and virus-free SBPH used throughout the experiment were fed at the Institute of Plant Protection in JAAS. Young instar nymphs of SBPH were maintained on healthy rice seedlings (*Oryza sativa L. cv.* Wuyujing No. 3) in an insect-rearing room at 25 °C until infestation. The rate of viruliferous SBPHs in the virus-acquiring group was detected using a Dot enzyme-linked immunosorbent assay (Dot-ELISA) according to Zhou et al. [25].

The soil of 14-day-old rice seedlings was kept as dry as much as possible, which was followed by various treatments and placement in darkness for 12 h. Then, rice seedlings were inoculated with viruliferous nymphs according to the inoculation method from Zhou et al. for 3 days [26]. Subsequently, all insects were removed from the plants, which were transferred to the soil in the cement pool of greenhouse in the green house. In addition, virus-free SBPHs were used for the control group.

### The analysis of disease incidence of RSV

Thirty rice seedlings of each cultivar were tested for disease incidence experiment. At 30 days post inoculation, the amount of symptomatic plants was counted, and the disease incidence of RSV is the percentage of the symptomatic plants in total 30 plants.

### Quantitative analysis of melatonin

Approximately 100 mg rice leaves inoculated with RSV were harvested and immediately ground under liquid nitrogen; the powder was extracted in 900 μL of 10 mM PBS (phosphate buffered saline) buffer (pH 7.2) and centrifuged at 12,000 *g* for 10 min, the supernatant was prepared for assay. A melatonin detection ELISA kit, purchased from Jiangsu Bao Lai Biological Technology Co., Ltd. China, was used to quantify the endogenous melatonin, following the manufacturer’s instructions. All experiments were repeated at least three times.

### Quantification of NO content by laser scanning confocal microscopy

Cross sections (3 mm thick) were cut from stems of viruliferous or nonviruliferous SBPH-inoculated rice plants, infiltrated with an NO fluorescent probe (10 μM 4-amino-5-methyl-amino-2′,7′-di-fluorofluorescein diacetate [DAF-FM DA]) diluted in a 20 mM Hepes-NaOH buffer, pH 7.2, followed by 15 min incubation in the dark [27, 28].

After thoroughly rinsing in the Hepes-NaOH buffer, the sections were examined, imaged, and processed using a Zeiss LSM 710 confocal laser scanning microscope equipped with ZEN software (Carl Zeiss, Oberkochen, Germany). The excitation wavelength was set at 488 nm, and the emission wavelength was set at 500–530 nm. More than 10 rice plants were analyzed for each treatment. The average of fluorescence intensity for each treatment was shown in the graph.

### RNA isolation and quantitative real-time PCR

Total RNAs from 100 mg of rice leaves were extracted with Trizol reagent (Invitrogen, Gaithersburg, MD, USA) according to the manufacturer’s instructions, and then they were dissolved in DNase-treated distilled water. The concentration and quality of isolated RNA were determined using a NanoDrop 2000 spectrophotometer (Thermo Fisher Scientific, Wilmington, DE, USA). Next, cDNA was synthesized from 1 μg of total RNA using an oligo (dT) primer and MMLV reverse transcriptase (BioTeke, Beijing, China). RT-qPCR was performed using SsoFastTM Eva Green® Supermix (Bio-Rad, Shanghai, China) with the Bio-Rad iQ5 RT-qPCR system. Multiple internal reference genes, *UBQ10* and *GAPDH*, were used in this assay [29]. All primers used in this experiment are shown in Additional file 1: Table S1. The 2^-ΔΔCt^ method [30] was used to calculate relative expression levels.

### Statistical analysis

All experiments were repeated three independent times and achieved similar results. Data represent the means ± SD of triplicate measurements. Statistical analysis was performed using SPSS 18.0 and Duncan’s multiple tests, and *P* < 0.05 was considered as significant different and indicated with a different letter or asterisk.

## Results

### Zhendao 88 is resistant to RSV infection

**Fig. 1:**
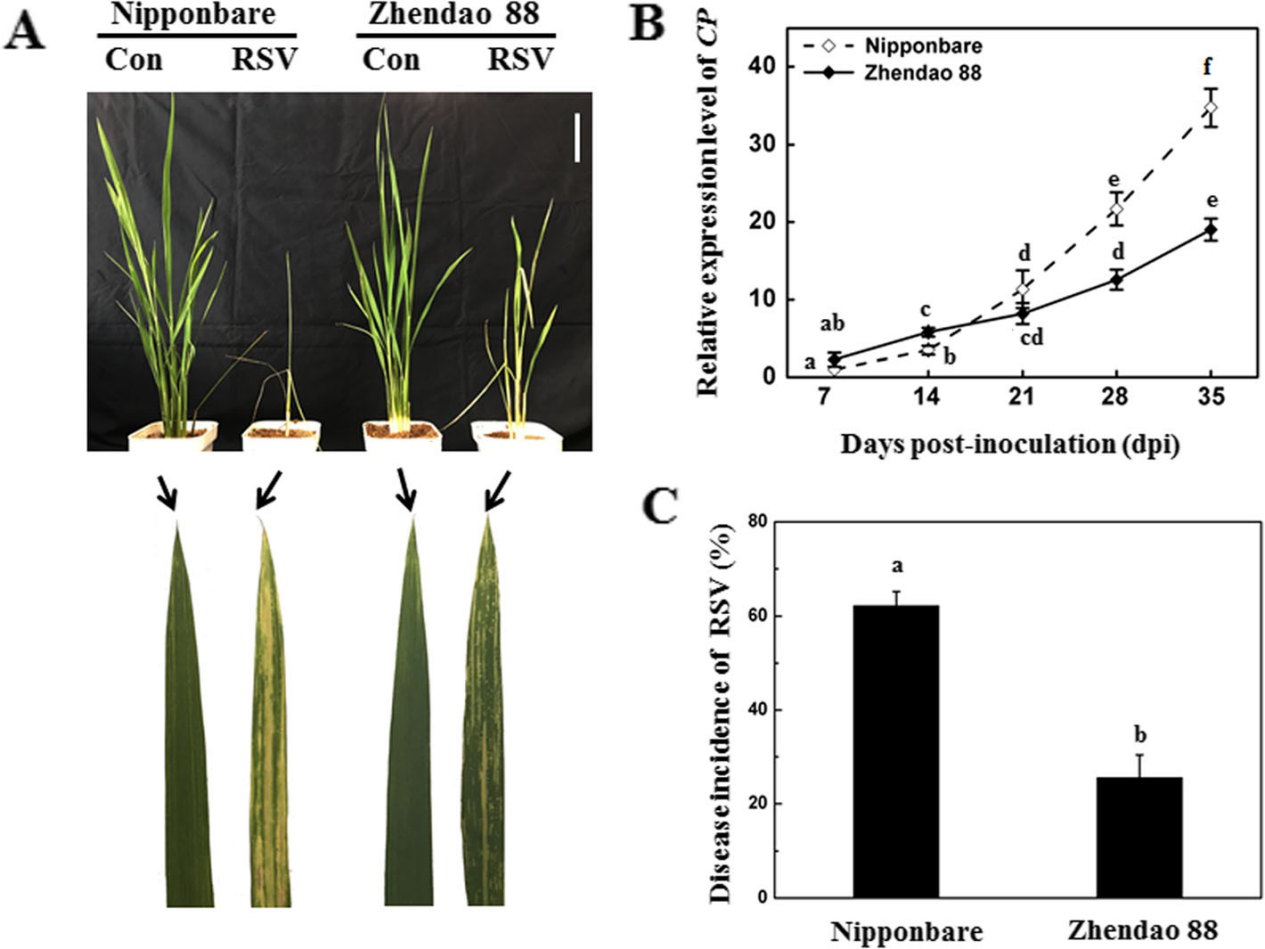
Different phenotypes of Nipponbare (susceptible) and Zhendao 88 (resistant) inoculated with RSV. **a** Symptoms of the two varieties at 28 dpi. The lower panel of (**a**) shows the symptomatic leaves of the representative plants. Bars = 5 cm. **b** Relative expression of RSV *CP* in the two varieties at 7, 14, 21, 28 and 35 dpi. **c** Disease incidences of the two varieties at 30 dpi. Thirty plants were used for each treatment in the experiment of disease incidence. All the experiments were repeated three times, and similar results were obtained. The data represent the means ± SD of triplicate measurements. Different letters represent significantly difference at *P* < 0.05 according to Duncan’s multiple tests

To detect the relationship of MT and NO with resistance to RSV, we first verified the different phenotypes presented by Nipponbare and Zhendao 88, which were susceptible and resistant to RSV, respectively. As shown in Fig. 1, compared to the control group, Nipponbare had more severe symptoms than Zhendao 88 (Fig. 1a). The expression level of *CP* in the two rice varieties was assayed after RSV infection. Quantitative RT-PCR using RSV *CP* specific primers showed that RSV RNA accumulated similar in both RSV-inoculated Zhendao 88 and Nipponbare plants at 7, 14 and 21 dpi, but RSV RNA accumulated in Zhendao 88 plants was much lower than in Nipponbare plants at 30 dpi the expression level of *CP* was significantly higher in Nipponbare than Zhendao 88 at 28 and 35 dpi (Fig. 1b). Consistently, approximately 85% of the RSV-inoculated Nipponbare plants showed virus symptoms, while only approximately 10% of the RSV-inoculated Zhendao 88 plants showed virus symptoms (Fig. 1c). Thus, these two rice cultivars were used for further analysis.

### The endogenous MT content increased in resistant cultivar Zhendao 88 after RSV infection

**Fig. 2:**
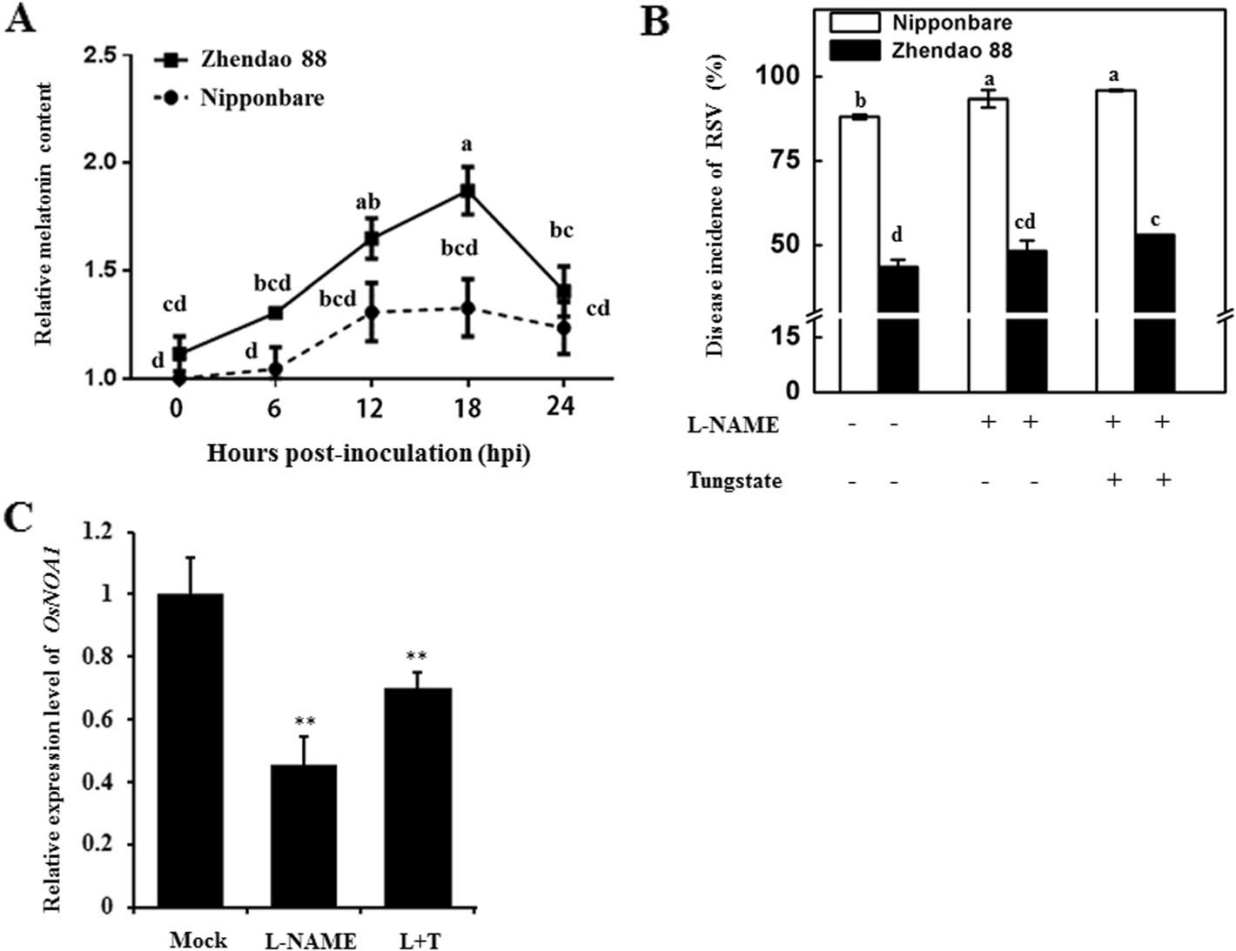
The endogenous MT content in rice plants after RSV infection. **a** Relative endogenous MT level of the two varieties in response to RSV pathogen infection within 24 h. The value of the control group was set at 1.0. **b** Disease incidence of the two varieties pretreated with 200 μM L-NAME (NOS inhibitor), or the combination of treatments with 200 μM L-NAME and 200 μM Tungstate (NR inhibitor). **c** Effect of inhibitors on the expressions of *OsNOA1* in Nipponbare plants under normal conditions. Mock: water treated. L-NAME: 200 μM L-NAME treated. L + T: 200 μM L-NAME and 200 μM Tungstate together. All the experiments were: repeated three times, and similar results were obtained. The data represent the means ± SD of triplicate measurements. Different letters or asterisks represent significantly difference at *P* < 0.05 according to Duncan’s multiple tests

To explore whether MT is responsible to rice resistance to RSV, we assayed the difference in MT content between Nipponbare and Zhendao 88, which were demonstrated to be susceptible and resistant to RSV, respectively, in Fig. 1. The time-course experiment showed that the relative MT content in Zhendao 88 was significantly higher than Nipponbare (Fig. 2a). In addition, in the inhibitor assay with L-NAME and Tungstate, NOS and NR inhibitors, disease incidence increased significantly with NOS inhibitor and inhibitors combination, indicating that NO was also responsible for the resistance to RSV infection (Fig. 2b). Under normal condition, qRT-PCR results showed that the relative expression level of *OsNOA1*, which was shown to be related to the NOS-dependent pathway [31, 32], was significantly lower in the L-NAME-treated and Tungstate plus L-NAME-treated plants (Fig. 2c). The results suggested that NO content indeed decreased following applications of these two inhibitors. All the results in Fig. 2 were a preliminary indication of the positive role of MT and NO in the response of rice to RSV infection.

### The resistance to RSV can be improved by increased MT through a NO-dependent pathway

**Fig. 3:**
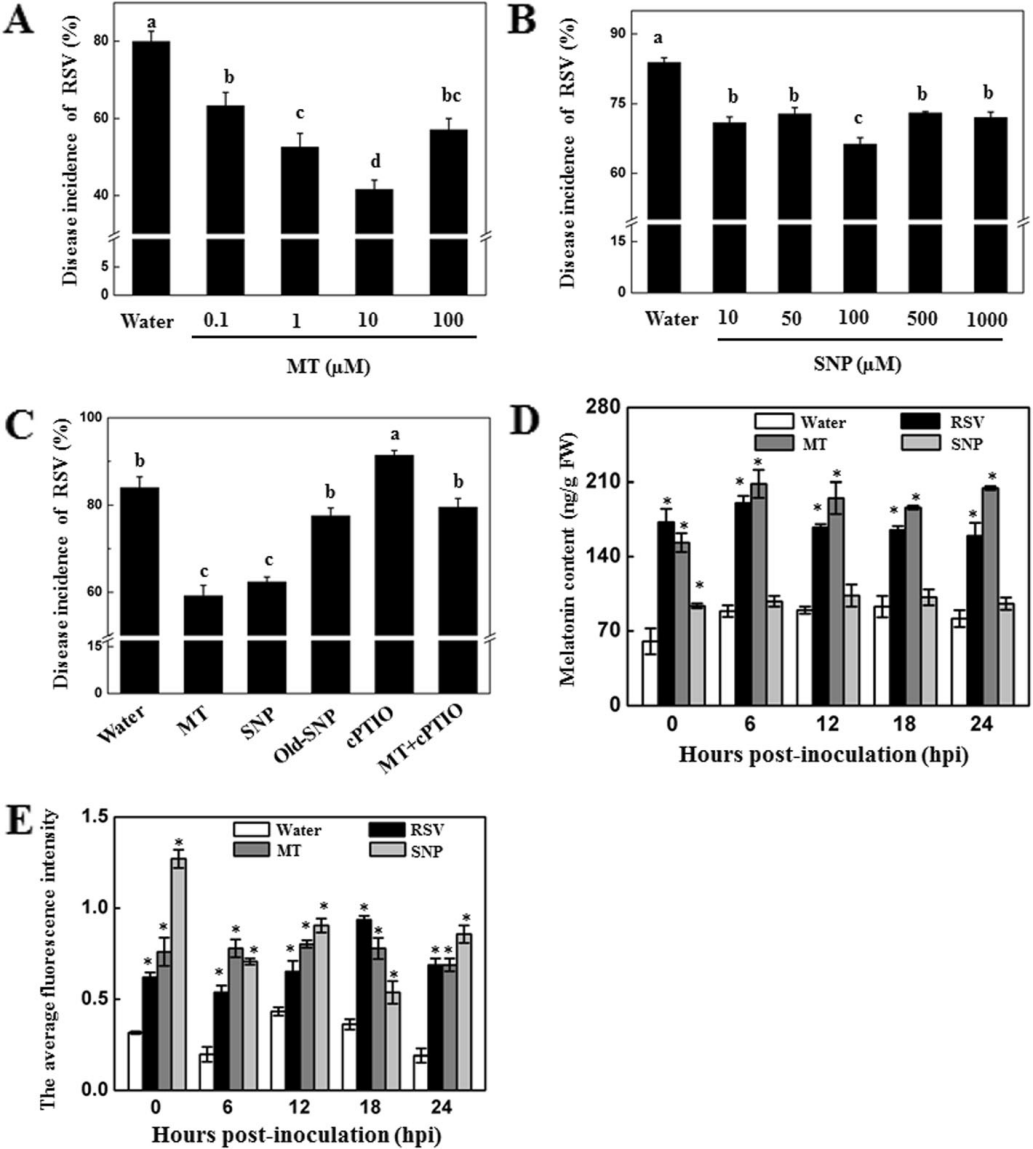
The resistance to RSV can be improved by the increased production of MT and NO. The high disease incidence of Nipponbare inoculated with RSV can be reduced by exogenous MT and NO. The disease incidence of Nipponbare was recorded at 30 dpi pretreated with: (**a**) The disease incidence of Nipponbare inoculated with RSV pretreated with four different concentrations of MT (0.1, 1, 10 and 100 μM) or (**b**) with five different concentrations of SNP (10, 50, 100, 500 and 1000 μM) for 12 h and inoculated with viruliferous for 3 d. **c** The disease incidence of Nipponbare pretreated with deionized water, 10 μM MT, 100 μM SNP, 100 μM Old-SNP, 100 μM cPTIO and 10 μM MT + 100 μM cPTIO for 12 h and then inoculation with RSV for 3 d. All data were recorded at 30 dpi. Thirty plants were used for each treatment in the experiment of disease incidence. **d** and (**e**) Nipponbare plant treated with virus-free SBPH, RSV (viruliferous SBPH), 10 μM MT or 50 μM SNP. The rice plants were inoculated by virus-free SBPH or viruliferous SBPH for 3 days, then all SBPHs were removed from the plants, the time point when SBPHs were removed was set as 0 hpi. After 0, 6, 12, 18 and 24 h, plants were taken immediately for the melatonin or NO assays. All the experiments were repeated three times, and similar results were obtained. The data represent the means ± SD of triplicate measurements. Different letters or asterisks represent significantly difference at *P* < 0.05 according to Duncan’s multiple tests

To further analyze the function of MT and NO in the response of rice to virus, Nipponbare plants were pretreated with exogenous MT and NO. We initially screened the optimum concentration of MT and SNP, a specific NO donor. The results showed that MT and SNP could both reduce the disease incidence in a concentration dependent manner, with the largest effects at 10 μM of MT and 100 μM of SNP, resulting in 30.00 and 25.78% disease incidence reduction, respectively (Fig. 3a and b). Thus, 10 μM MT and 100 μM SNP were selected for the follow-up experiment. Moreover, 100 μM Old-SNP and 100 μM cPTIO were also pretreated in the inoculation experiment. As shown in Fig. 3c, compared to the water-treated group, Old-SNP treatment failed to reduce the disease incidence as SNP did, and cPTIO can exacerbate disease, which indicated that NO may play a vital role in the rice response to RSV infection. In addition, cPTIO could eliminate the MT positive effect on the disease incidence, which suggested that NO serves as a mediator in MT triggered rice resistance to RSV infection. Overall, we speculated that MT and NO can promote rice resistance to RSV, and NO functions downstream of MT in this pathway.

To further detect the participation of MT and NO in rice-virus interactions, we assayed the production of MT and NO in rice plants after RSV infection firstly. The result showed that MT and NO production were accumulated higher in RSV-inoculated rice plants than water-treated virus-free inoculated plants, which indicated that the MT and NO production might be induced in rice-RSV interactions. Results of the MT and NO treatments experiments showed that MT-treated rice plants accumulated about more MT and NO compared with water-treated virus-free inoculated plants, while SNP-treated rice plants accumulated more NO but not MT (Fig. 3d and e). Taken all results in Figs. 3 together, it demonstrated that MT and NO can respond to RSV infection, and the effect of MT on rice resistance to RSV is through a NO-dependent pathway.

### Expression patterns of *OsPR1b* and *OsWRKY 45* after various treatments

**Fig. 4:**
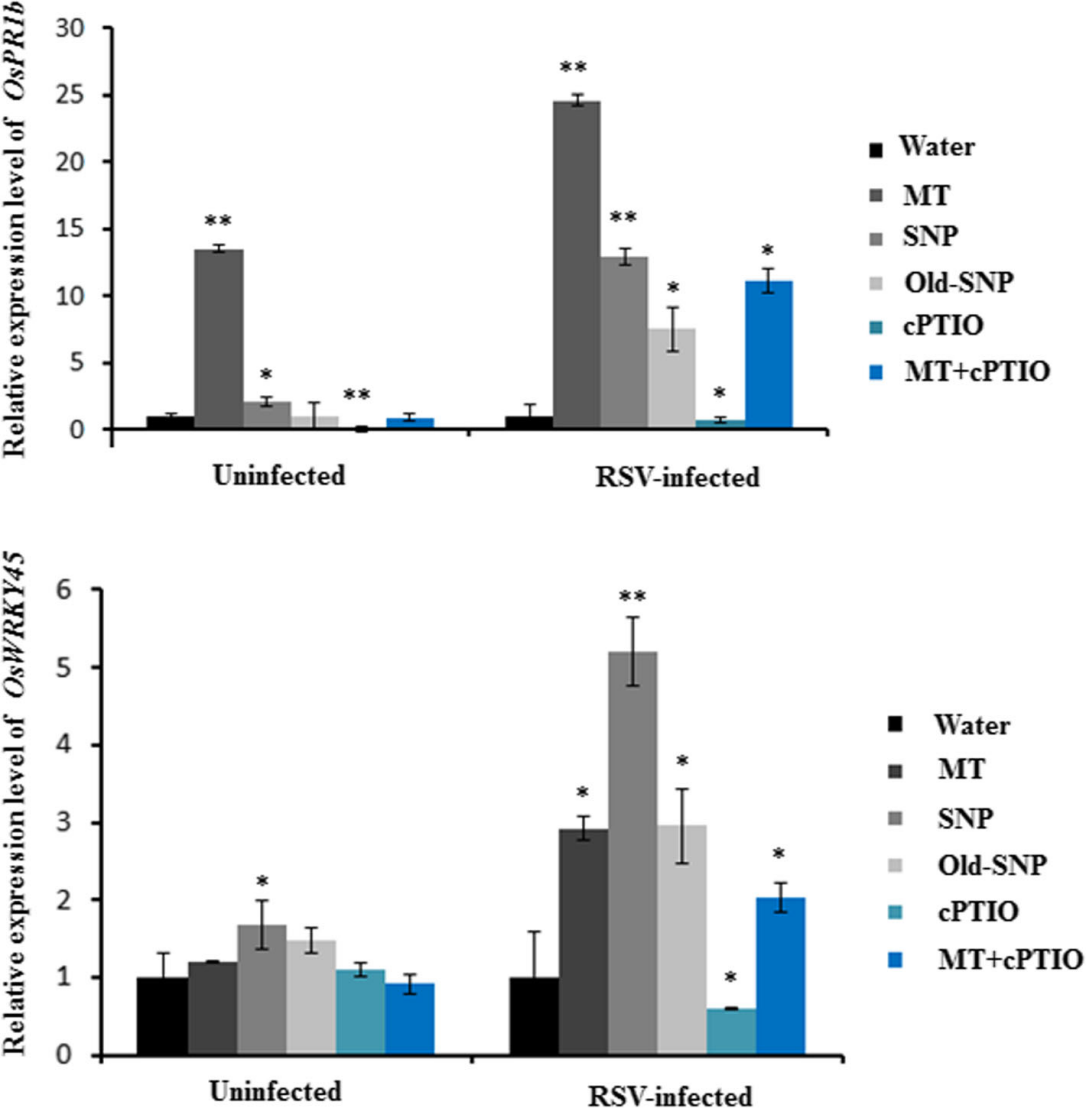
Transcriptional profiles of *OsPR1b* and *OsWRKY 45*. Nipponbare plants were pretreated with different treatments and then were inoculated with virus-free SBPH or RSV (viruliferous SBPH). Total RNA was extracted from rice leaves at 18 hpi. The assay was performed by qPCR and normalized against the combination of *UBQ 10* and *GAPDH*. The values of the water-treated group were set at 1.0. All the experiments were repeated three times, and similar results were obtained. The data represent the means ± SD of triplicate measurements. Different letters or asterisks represent significantly difference at *P* < 0.05 according to Duncan’s multiple tests

Since *OsWRKY 45* was reported to play an essential role in rice blast resistance [33] and it is well-known that induced expression of PR genes is able to enhance the plant’s disease-resistance [34], the expression levels of *OsPR1b* and *OsWRKY 45* in rice plants with various treatments were analyzed at 18 hpi using quantitative real-time PCR. Compared to the water-treated group, the two genes were induced by both MT and SNP treatment and suppressed by cPTIO significantly after RSV infection, while the expression levels of *OsPR1b* was also induced by MT and SNP treatments, and *OsWRKY 45* was induced by SNP treatment in uninfected plants, although the inducible level is lower than that in RSV infected plants (Fig. 4). The results indicated that *OsPR1b* and *OsWRKY 45* were induced by RSV infection and *OsPR1b* might act downstream of MT and NO signaling pathway, and *OsWRKY 45* could be partially downstream of MT and NO signaling pathway, because of the low expression level after the treatments in uninfected plants. Interesting, Old-SNP and the combination of MT and cPTIO could also up-regulate their expression, although the change was relatively minor; thus, further study is needed to determine whether they function in other pathways.

## Discussion

Because of the lack of effective protection against rice damage caused by RSV infection and the concerns about the safety of transgenic technology, exogenous applications of protective biological treatments may be acceptable and environmentally friendly [35]. Although some studies have shown that MT and NO can improve the resistance of plants to pathogens, their effect on rice-virus interactions remains elusive [11, 13, 36]. Recently, the association between MT and NO in disease resistance has been reported not only in animals but also in plants [13, 37]. Consistent with these studies, we found that NO participates in MT triggered rice resistance to RSV.

The first evidence is that MT can be significantly induced in resistant cultivar in response to RSV infection (Fig. 2a), and accordingly, it correlated with high levels of rice resistance to RSV (Fig. 1c). These results preliminarily suggested the protective role of MT to RSV stress. Secondly, pharmacological experiments with both exogenous MT and NO alleviated the disease incidence of RSV, while application with cPTIO had a negative effect on disease incidence, which indicates that MT and NO can improve rice resistance against the RSV pathogen (Fig. 3). Overall, our results demonstrated the vital role of MT and NO in the rice-RSV interaction.

Strikingly, we found that cPTIO can reverse the positive effect of MT on disease incidence, which led us to deduce that NO is a mediator in the process of MT triggered rice resistance to RSV. Moreover, MT application can significantly enhance NO content level, while a NO donor has no effect on endogenous MT levels (Fig. 3). These results further indicated that NO is a downstream signal component of MT triggered rice resistance to RSV, which is consistent with the previous report of Shi et al. on *Arabidopsis* [13]. However, this mode of action is not always the case, because Pozo et al. and Aydogan et al. both reported the inhibitory effect of MT on biosynthesis of NO [38, 39]. Thus, the detailed mechanism of the concentration-dependent interaction between MT and NO in plant-pathogen interactions needs to be studied further.

According to the previously article [40], the rice variety Zhendao 88 did carry a resistant allele of *STV11*, which encoded a sulfotransferase that catalyzes the conversion of salicylic acid into sulphonated SA, confers resistance in RSV. It has also been reported that NO is required for the full function of SA [41], we speculated that the increase of MT in Zhendao 88 could lead more accumulated NO, which might lead to more SA. So sulphonated SA may be increased by the conversion of accumulated SA, which may be the reason why Zhendao 88 is resistant to RSV.

*Oryza sativa* WRKY transcription factor, *OsWRKY 45*, was reported to promote systemic acquired resistance (SAR) independent of *OsPR1* [33, 42], although not all of the WRKY genes reported are positive during pathogenesis [43]. Therefore, we further assayed the relative expression of *OsPR1b* and *OsWRKY 45*, as shown in Fig. 4; it was demonstrated that both MT and SNP application positively modulated these two genes, which confirmed our results described above.

In conclusion, MT is responsible for rice resistance to RSV infection by inducing NO. However, not only gas signaling molecules and plant hormones but also other components, such as carbohydrate and alcohols, also participate in the MT triggered plant resistance to pathogen infection [44]. Thus, it may have a broad application prospect on the further in-depth study of MT in plant-pathogen interactions.

## Supplementary information

**Additional file 1: Table S1.** Primers used for quantitative reverse transcription polymerase chain reaction (qRT-PCR).

## Data Availability

All data generated or analyzed during this study are included in this published article.

## Abbreviations

CP: Coat protein
cPTIO: 2-(4-carboxyphenyl)-4, 4, 5, 5-tetramethylimidazoline-1-oxyl-3-oxide
MT: Melatonin
NO: Nitric oxide
NOS: NO synthase
NR: nitrate reductase
RSV: *Rice stripe virus*
SBPH: Small brown planthopper
SNP: a NO-releasing reagent
