# Supplementary material for: Melatonin is responsible for rice resistance to rice stripe virus infection through a nitric oxide-dependent pathway
Source: Virol J. 2019 Nov 21;16:141. doi: 10.1186/s12985-019-1228-3 (PMC6869260; doi:10.1186/s12985-019-1228-3)
Supplement: Supplementary file 1 — Additional file 1: Table S1. Primers used for quantitative reverse transcription polymerase chain reaction (qRT-PCR). [file 12985_2019_1228_MOESM1_ESM.docx]

**Table S1.** Primers used for quantitative reverse transcription polymerase chain reaction (qRT-PCR)

| Gene name | Accession number | | Primer sequence (5′→3′) |
| --- | --- | --- | --- |
| *UBQ 10* | | AK101547 | F-TGGTCAGTAATCAGCCAGTTTGG  R-GCACCACAAATACTTGACGAACAG |
| *GAPDH* | | AK064164 | F-AAGCCAGCATCCTATGATCAGATT  R-CGTAACCCAGAATACCCTTGAGTTT |
| *CP* | | NC_003776.1 | F-TGCAGAAGGCAATCAATGACAT  R- TGTCACCACCTTTGTCCTCAA |
| *OsNOA1* | | LOC4328006 | F-GAGACACCATCAGCTGAGAACCGTG  R-CATGACGAAGGTAGGAGAGCTTGTC |
| *OsPR1b* | | AK107926 | F-ACGCCTTCACGGTCCATAC  R-AAACAGAAAGAAACAGAGGGAGTAC |
| *OsWRKY 45* | | EF143611 | F-TCAGTGGAGAAGCGGGTGGTG  R-GGGTGGTTGTGCTCGAAGGAG |
